# Reinforcement of Dextran Methacrylate-Based Hydrogel, Semi-IPN, and IPN with Multivalent Crosslinkers

**DOI:** 10.3390/gels10120773

**Published:** 2024-11-27

**Authors:** Luca Paoletti, Gianluca Ferrigno, Nicole Zoratto, Daniela Secci, Chiara Di Meo, Pietro Matricardi

**Affiliations:** Department of Drug Chemistry and Technologies, Sapienza University of Rome, P.le A. Moro 5, 00185 Rome, Italy; luca.paoletti@uniroma1.it (L.P.); gianluca.ferrigno@uniroma1.it (G.F.); nicole.zoratto@pharma.ethz.ch (N.Z.); daniela.secci@uniroma1.it (D.S.); chiara.dimeo@uniroma1.it (C.D.M.)

**Keywords:** dextran methacrylate, crosslinkers, hydrogel, IPN, stiffness

## Abstract

The need for new biomaterials to meet the needs of advanced healthcare therapies is constantly increasing. Polysaccharide-based matrices are considered extremely promising because of their biocompatibility and soft structure; however, their use is limited by their poor mechanical properties. In this light, a strategy for the reinforcement of dextran-based hydrogels and interpenetrated polymer networks (semi-IPNs and IPNs) is proposed, which will introduce multifunctional crosslinkers that can modify the network crosslinking density. Hydrogels were prepared via dextran methacrylation (DexMa), followed by UV photocrosslinking in the presence of diacrylate (NPGDA), triacrylate (TMPTA), and tetraacrylate (PETA) crosslinkers at different concentrations. The effect of these molecules was also tested on DexMa-gellan semi-IPN (DexMa/Ge) and, later, on IPN (DexMa/CaGe), obtained after solvent exchange with CaCl_2_ in HEPES and the resulting Ge gelation. Mechanical properties were investigated via rheological and dynamic mechanical analyses to assess the rigidity, resistance, and strength of the systems. Our findings support the use of crosslinkers with different functionality to modulate the properties of polysaccharide-based scaffolds, making them suitable for various biomedical applications. While no significative difference is observed on enriched semi-IPN, a clear improvement is visible on DexMa and DexMa/CaGe systems when TMPTA and NPGDA crosslinker are introduced at higher concentrations, respectively.

## 1. Introduction

Biomaterials have raised growing interest for their use in several biomedical and healthcare applications, representing a turning point in the scenery of advanced medical solutions, such as the diagnosis, treatment, and regeneration of damaged tissues and organs [1,2,3]. Biocompatibility, biodegradability, high water content, soft structure, and porosity are just some of the properties that make these matrices able to closely resemble living tissues, showing great potential in various fields, such as agriculture, the food industry, drug delivery, tissue engineering, and regenerative medicine [4,5,6,7]. Single-network hydrogels have been extensively used in biomedicine due to their ease of synthesis and cost-effectiveness. However, they often lack adequate mechanical properties, mostly because of inherent structural inhomogeneity and the absence of stress dissipation mechanisms to prevent crack propagation. Therefore, their practical utility is significantly hindered by their soft, fragile, and brittle nature [8,9]. Different strategies have been employed to improve biomaterials’ individual properties, including the development of highly entangled and amphiphilic networks [10,11]. In this light, multicomponent networks have been designed to create composite hydrogels, which include a minimum of two distinct polymers [12]. Among them, semi-IPN and IPN hydrogels notably enhance the mechanical strength of the biomaterial: defined as dual polymeric interpenetrated systems in which one (semi-IPN) or both (IPN) of the components is/are crosslinked independently from the other; they also provide an effective drug loading capacity and controlled swelling behavior [13,14]. The combination of a first network, usually brittle, rigid, and chemically crosslinked, which contributes to the strength and rigidity of the hydrogel, with a second one, often soft and weakly crosslinked, which absorbs external stress to enhance the hydrogel’s flexibility and toughness, results in a system with excellent mechanical properties, able to dissipate energy efficiently [15,16].

The incorporation of polysaccharides into IPN systems has become a popular research topic, as a strategy to overcome the poor mechanical properties of polysaccharide-based hydrogels whose applications are still hampered by this limitation [17,18,19]. Furthermore, the use of natural polymers contributes to improving a hydrogel’s biocompatibility and cell adhesiveness and to reducing toxicity and immunogenicity that can derive from toxic degradation products of synthetic materials [20,21]. The presence of functional groups on the polysaccharide’s backbone, such as hydroxy, carboxyl, acetyl, amino, and sulfonic acid groups, gives rise to potential gel formation through multiple interactions like hydrogen, coordination bonds, and electrostatic interactions [17,22]. Matrices crosslinking can be also promoted by further modification on such groups with the aim to introduce hydrophobic molecules or reactive groups that can establish inter- or intra-molecular interactions when exposed to internal or external stimuli [8,14]. In this view, functionalization of polysaccharide chains with methacrylate moieties allows obtaining derivatives that can be crosslinked by means of UV light, an easy, fast, and safe method that leads to stable and hard (semi)-IPN systems [23,24]. Dextran, gellan, hyaluronic acid, or alginate methacrylate hydrogel, semi-IPN, and IPN have been widely described as interesting platforms for tissue engineering and drug delivery applications [25,26,27]; however, to the best of our knowledge, the effect of the introduction of multifunctional crosslinkers on the crosslinking kinetics and the stiffness of the resulting matrices has never been investigated. Hydrogel prepared with conventional methods, indeed, have been characterized by spatial inhomogeneity, while it has been shown that the addition of crosslinking agents considerably increases the structural homogeneity of the networks, which results in different properties (swelling, pore structure, mechanical properties, etc.) [28,29]. In addition, the length of the crosslinker plays an important role in increasing or decreasing the homogeneity of the scaffold: homogeneous hydrogels were obtained using the large size and flexible dimethacrylate crosslinkers, increasing the distance between the vinyl groups of the molecule [30]. Finally, crosslinker functionality has, also, an impact: crosslinkers with high functionality could lead to networks containing a significant number of imperfections and defects, which affect the mechanical performance of the hydrogels [31].

In this work, we investigated the effect of the introduction of crosslinkers with different functionality into dextran-based, single-network hydrogel, and double-network IPN systems. Dextran (Dex) was functionalized with methacrylic groups (DexMa) and then photocrosslinked with UV light in the presence of diacrylate (NPGDA), triacrylate (TMPTA), or tetraacrylate (PETA) crosslinkers. Different irradiation times (1 and 5 min) and concentrations of crosslinkers (10:1, 4:1, and 2:1 ratios between moles of methacrylic units on the Dex backbone and moles of crosslinking molecules) have been tested. Furthermore, the impact of such molecules was also examined on DexMa/gellan gum (Ge) semi-IPN systems, varying the above-mentioned parameters. Both matrices were developed in glycerol, a non-conventional solvent which proved to speed up the kinetics of crosslinking, acting as a co-initiator in the process [32]. Then, DexMa/Ge semi-IPN underwent solvent exchange with HEPES buffer and CaCl_2_, which led to the conversion into IPN systems due to the gelation of the Ge chains with calcium ions. Mechanical properties of DexMa hydrogels, DexMa/Ge semi-IPN, and IPN both with and without crosslinkers have been evaluated, outlining the impact of multifunctional crosslinkers on improving biomaterial characteristics.

## 2. Results and Discussion

Derivates of propane-1,3-diol have been widely used as the building block for the synthesis of plastics, paints, and cosmetics [33,34,35]. Indeed, hydroxyl groups of such molecules can be functionalized with acrylic acid to obtain various acrylates, with different degrees of functionality, that can be used as monomers in the manufacture of polymers [36,37,38]. The presence of the acrylate group allows these compounds both to react with amines by means of a Michael addition reaction [39] and to form carbon–carbon bonds via radical reaction [40], which have been exploited in the drug delivery and tissue engineering fields [41,42,43]. Recently, their use as crosslinkers has been particularly beneficial in modulating the properties of scaffolds which lack suitable mechanical characteristics and stability, by influencing the density of crosslinking [44,45]. Dextran-based hydrogels and semi-IPN systems have proved to be excellent candidates for different biomedical applications [46,47,48], also based on their biodegradability. However, since most of them suffer from several mechanical disadvantages (i.e., low resistance to stresses, brittleness, low deformability, etc.), numerous strategies have been developed to overcome these limitations [49,50]. In this light, the addition of crosslinkers could promote the formation of more homogeneous, resistant, and stable matrices. For that reason, we evaluate the impact of multifunctional crosslinkers on the mechanical properties of dextran methacrylate (DexMa)-based hydrogels, semi-IPNs, and IPNs. Diacrylate (NPGDA), triacrylate (TMPTA), and tetraacrylate (PETA) propane-1,3-diol derivatives have been tested, by varying concentrations and time of exposure to UV light, to assess how the stiffness of the scaffolds could be related to different crosslinking densities. For the sake of completeness, it would be also necessary to discuss the potential effect that the introduction of these crosslinkers could have on the biodegradability of the matrices. Indeed, the biodegradability of dextran-based systems has been widely discussed in the literature [51,52], but it has also been recognized that the degradation time can be modulated from 1 day to three months depending on the crosslinking density and, consequently, the type of crosslinker used [53]. This work is particularly focused on the mechanical characterization of such systems, but the biodegradability of matrices containing NPGDA, TMPTA, and PETA will be tested in the future to select the most suitable crosslinker for applications requiring different matrix erosion times.

### 2.1. DexMa Hydrogels and DexMa/Ge Semi-IPNs Preparation

**Figure 1:**
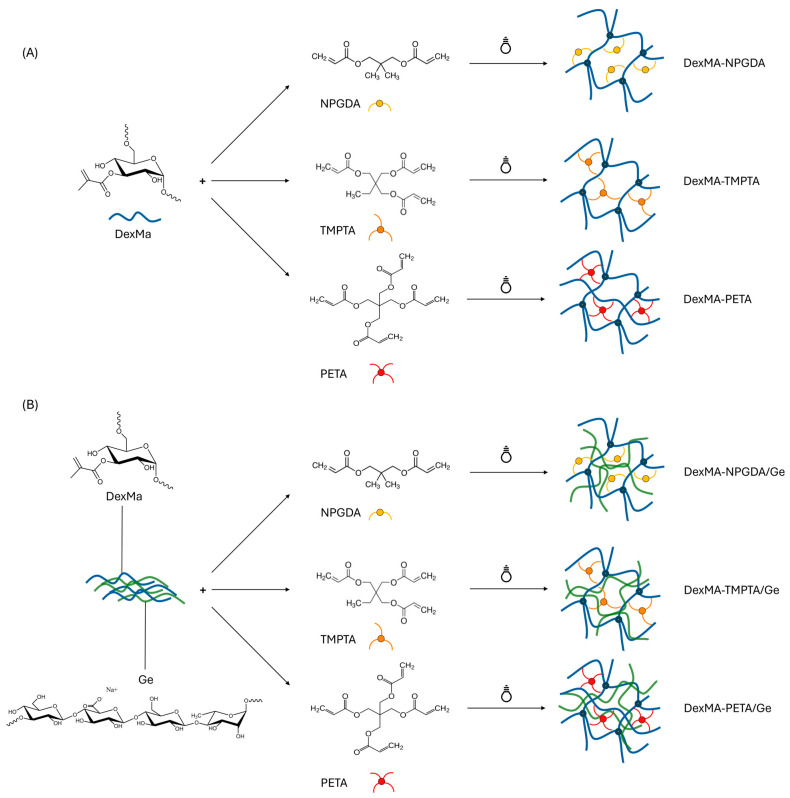
Scheme of DexMa hydrogels’ (**A**) and semi-IPNs’ (**B**) formation, in the presence of diacrylate, triacrylate, and tetraacrylate crosslinkers, after UV photocrosslinking.

DexMa derivative was characterized via NMR to assess the degree of methacrylation: as expected, a DS of 20% mol/mol was obtained, confirming the success of the reaction (Figure S1 in Supplementary Materials). Therefore, both hydrogels and semi-IPN systems were obtained by solubilizing DexMa and Ge in glycerol, according to the procedures reported in Section 4.2.2 and Section 4.2.3. The use of high temperatures allowed for the obtainment of clear and homogeneous solutions, without polysaccharide chain degradation [32]. After solubilization, crosslinkers were added dropwise in different concentrations and ratios. First, the solubility of the water-insoluble neopentyl glycol diacrylate (NPGDA), trimethylolpropane triacrylate (TMPTA), and pentaerythritol tetraacrylate (PETA) was tested in DMSO and N-vinyl-2-pyrrolidone at a concentration of 500 μg/mL. Then, the obtained solutions were added to glycerol to test the miscibility and the minimal quantity of organic solvent to add to have a stable and homogeneous mixture. DMSO performed better than N-vinyl-2-pyrrolidone and, therefore, solutions of NPGDA, TMPTA, and PETA were prepared at different concentrations to maintain the same amount of organic solvent introduced into the unaltered system (13% *w*/*w*). Finally, the irradiation of the mixture with UV light allowed the crosslinking between the methacrylic groups of the DexMa and the crosslinkers, leading to the formation of a chemical network (Figure 1A). In the case of semi-IPNs, the crosslinking took place in the presence of Ge, which is interspersed into the DexMa network (Figure 1B) [32].

Due to its capacity to perform as a radical co-initiator, reducing the concentration of photoinitiator needed and speeding up the crosslinking kinetics, glycerol contributes significantly to the process [32]. It is widely known, indeed, that chain crosslinking is facilitated by glycerol’s ability to act as a multifunctional chain transfer agent at sufficiently high concentration [54].

### 2.2. Hydrogel Dynamic Mechanical Characterization

The effect of the presence of crosslinkers on DexMa hydrogels’ mechanical properties was evaluated by testing the response of the matrices when subjected to uniaxial stresses, measuring the compressive strength (Young’s modulus; E) and shear forces and determining the shear storage and shear loss moduli (G′, G″) [55]. The stiffness of such hydrogels was first assessed with a compression experiment, in which the samples were placed on a roughened surface (to avoid slippage phenomena) and perpendicularly penetrated with a cylindrical probe. The applied force was correlated with the penetration depth, and E was calculated by extrapolating the tangent line in the first linear section of the curve.

**Figure 2:**
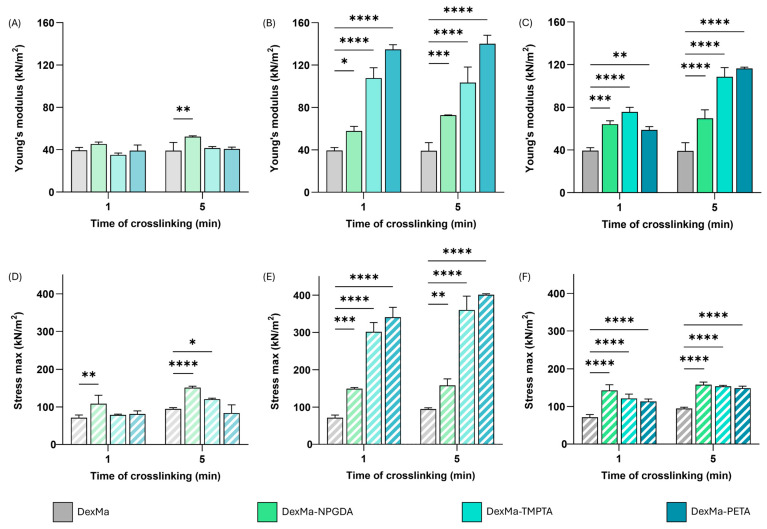
Dynamic mechanical analysis of DexMa systems. Young’s modulus [E] of DexMa, DexMa-NPGDA, DexMa-TMPTA, and DexMa-PETA hydrogels in a ratio of 10:1 (**A**), 4:1 (**B**), and 2:1 (**C**). Maximum stress registered at the breaking point of DexMa, DexMa-NPGDA, DexMa-TMPTA, and DexMa-PETA hydrogels in a ratio of 10:1 (**D**), 4:1 (**E**), and 2:1 (**F**). Data are expressed as the mean value ± standard deviation; experiments were performed in triplicate (*n* = 3). *p* values were obtained via two-way ANOVA and Dunnett’s multiple comparisons test (**** *p* < 0.0001; *** *p* < 0.001; ** *p* < 0.01; * *p* < 0.05).

Comparing the E values obtained from the analysis of the DexMa hydrogels to which the crosslinkers have been added with a ratio of 10:1 (Figure 2A), it is noted that the presence of NPGDA, TMPTA, or PETA has not contributed to the rigidity of the system. The modules are almost identical for all crosslinking times tested and, in any case, comparable with the control samples. This result is probably due to the reduced concentration of crosslinkers in the mixture, which, when evenly distributed in the system, is not enough to influence the crosslinking of the matrices. On the other hand, considering the hydrogels prepared with a 2:1 ratio (Figure 2C), it is possible to highlight an increase in the rigidity of the systems, compared to the control, as a function of the type of crosslinker and photocrosslinking time. In fact, while DexMa and DexMa_2_-NPGDA_1_ systems reach a constant Young’s modulus after only one minute of irradiation (∼40 kPa and 65 kPa, respectively), DexMa_2_-TMPTA_1_ hydrogels showed an E increase of 2× and 2.7× after 1 and 5 min of crosslinking, respectively. Surprisingly, the E of DexMa_2_-PETA_1_ matrices was lower than that of DexMa_2_-NPGDA_1_ and DexMa_2_-TMPTA_1_ after 1 min of crosslinking; however, an increase of 3×, compared to the control, was observed after 5 min of UV exposure. This trend, which was not significant in the hydrogels prepared with a ratio of 10:1, probably due to too low crosslinker concentration, seems to support the idea that adding crosslinkers with different crosslinking points leads to systems with different structures and, therefore, mechanical properties. In this case, an increase in the rigidity of the systems could be observed with the increase in the number of methacrylic groups in the crosslinker.

Given the significant difference between the samples prepared with the two ratios of 10:1 and 2:1, an intermediate ratio of 4:1 was also tested to prove a correlation between the concentration of crosslinkers in the system and the variation in its mechanical properties (Figure 2B). The E values of the resulting hydrogels were higher than those of the systems prepared with a ratio of 2:1, demonstrating that the previously assumed direct correlation does not exist. In particular, DexMa_4_-TMPTA_1_ and DexMa_4_-PETA_1_ matrices exhibit an E value 2.7- and 3.5-times higher than the value recorded without a crosslinker. In addition, the effect of the photocrosslinking time is not significant: a single minute of exposure to UV light is enough to give the hydrogel a stiffness higher than the control. A possible explanation could be related to the lower crosslinker concentration which might allow the establishment of shorter intra- and inter-chain bridges between DexMa chains, resulting in a tighter and consequently more rigid mesh system. Moreover, the fact that these properties are acquired after only one minute of crosslinking could be extremely advantageous from the perspective of 3D printing of scaffolds [56].

Observing the profile of the force applied by the probe as a function of deformation, it is possible to notice that there is an increasing trend, with increasing slope, as the distance covered by the probe increases, up to the breaking point of the gel, evidenced by a collapse of the curve. By focusing the attention at that point, it was possible to calculate the maximum force that the system was able to resist, which, compared to the unit of surface, gives a measure of the maximum stress that the system can bear. The percentage of deformation at which the gel rupture occurs was calculated, as reported in Table 1.

According to the previous results, the maximum stress values of the hydrogels obtained with a ratio of 10:1 are similar to those of the reference system (Figure 2D). This confirms that in such conditions the crosslinkers are not able to contribute to the formation of new inter- and intra-chain bridges such as to modify the resistance of the material to breakage. In contrast, DexMa samples with crosslinkers in a 2:1 ratio showed a slight increase in the max stress values as the photocrosslinking time increases (Figure 2F). Finally, the introduction of crosslinkers in a 4:1 ratio seems to have a significant impact on system resistance, resulting in a 4-fold increase in maximum stress for DexMa_4_-TMPTA_1_ and 5-fold for DexMa_4_-PETA_1_ (Figure 2E).

However, the percentage of deformation at which the break occurs is almost the same for all the tested ratios (see Table 1), except for DexMa-NPGDA hydrogels, which seem to withstand a deformation of almost 60%.

### 2.3. Rheological Characterization of Hydrogels

Mechanical properties of the hydrogels were then evaluated via a frequency sweep analysis, performed under stress-controlled conditions, using a grained plate–plate geometry to reduce the wall slippage phenomena. The mechanical spectra, in which the values of G′ and G″ are recorded as a function of frequency at 25 °C (Figure S2 in Supplementary Materials), reveal that all the systems have a gel-like behavior, with G′ > G″ in the entire frequency range (four decades). By extrapolating the value of G′ at the frequency of 1 Hz, it is possible to compare the elastic behavior of the various samples with that of the control and to evaluate the influence of the crosslinkers on G′ as a function of the crosslinking time. The analyses were performed after 1 or 5 min of UV irradiation. The experiments were carried out in triplicate.

**Figure 3:**
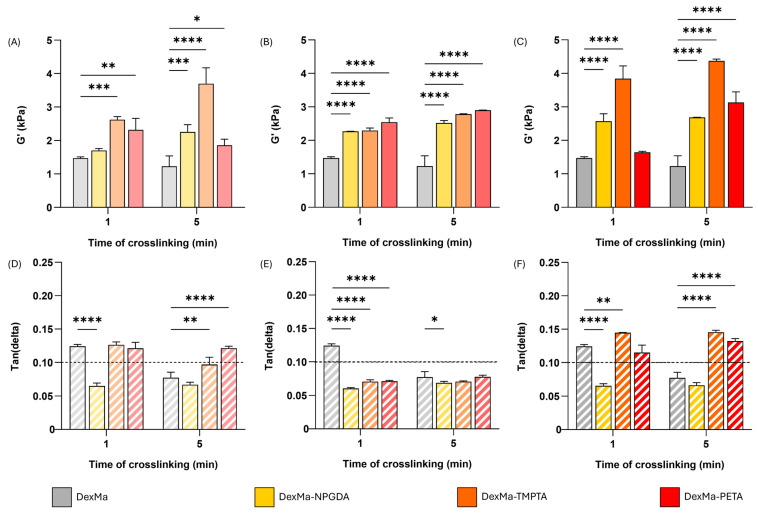
Hydrogel rheological analysis, showing the shear storage modulus (G′) at a frequency of 1 Hz, of DexMa, DexMa-NPGDA, DexMa-TMPTA, and DexMa-PETA prepared with a molar ratio of 10:1 (**A**), 4:1 (**B**), and 2:1 (**C**). Tan(delta) loss factor (G″/G′) at 1 Hz of hydrogels obtained with a ratio of 10:1 (**D**), 4:1 (**E**), and 2:1 (**F**). Data are expressed as the mean value ± standard deviation; experiments were performed in triplicate (*n* = 3). *p* values were obtained via two-way ANOVA and Dunnett’s multiple comparisons test (**** *p* < 0.0001; *** *p* < 0.001; ** *p* < 0.01; * *p* < 0.05).

In Figure 3A, G′ values at 1 Hz for DexMa systems with a crosslinker ratio of 10:1 are reported. Regarding DexMa_10_-NPDGA_1_ and DexMa_10_-TMPTA_1_ hydrogels, it is evident that the higher the photocrosslinking time, the higher the G′ value, which is always higher than the control. On the contrary, for DexMa_10_-PETA_1_, the exposure to UV light for 5 min does not seem to be advantageous. At higher crosslinker concentration, a significant increase in G′ was observed compared to the control for both photocrosslinking times (Figure 3B). However, while DexMA_4_-NPGDA_1_ and DexMa_4_-PETA_1_ showed a higher G′ absolute value compared to the one obtained with a 10:1 ratio, crosslinking of DexMa_4_-TMPTA_1_ surprisingly resulted in weaker hydrogels with respect to the one prepared with a lower ratio. Finally, testing the higher crosslinker ratio, a clearer growing trend was found (Figure 3C). In fact, DexMa_2_-NPGDA_1_, DexMa_2_-TMPTA_1_, and DexMa_2_-PETA_1_ hydrogels exhibit a G′ value that increases with the photocrosslinking time. Focusing on the hydrogels obtained after only 1 min of exposure to UV light, it is interesting to notice a significant increase in G′ compared to the control: 1.7-fold and 2.6-fold for DexMa_2_-NPGDA_1_ and DexMa_2_-TMPTA_1_, respectively. This is extremely advantageous in the light of 3D printing of the material, as this technique requires that the bioink is immediately and efficiently crosslinked after the extrusion so that the scaffold does not collapse on itself [57]. On the other hand, one-minute photocrosslinking of DexMa_2_-PETA_1_ hydrogels does not show any improvement in terms of the stiffness of the system.

Therefore, considering the rheological properties of DexMa hydrogels, it is possible to conclude that the addition of NPGDA and TMPTA is advantageous, compared to PETA, because it allows for the obtainment of high G′ values after 1 and 5 min of crosslinking. PETA still contributes to the cross-linking of the systems, but the crosslinking kinetics seem to be slower and more irregular, so it is difficult to find a correlation between elastic modulus and photocrosslinking time. This could be due to more acrylate functions of PETA than TMPTA and NPGDA, which give the system a higher order of complexity [31]. Furthermore, among all the molar ratios considered, 2:1 is the one that turned out to be the most advantageous as it allows a significant G′ increase even after short exposure times to UV light.

The rigidity achieved by hydrogels can be quantified using the tan(delta) parameter, obtained via the G″/G′ ratio [58]. Lower tan(delta) values are associated with higher stiffness; therefore, viscous liquid materials present tan(delta) ≥ 1, while gel structured systems show tan(delta) < 1. Bearing in mind that a gel can be classified as strong if its value falls below 0.1 [59], tan(delta) values at 1 Hz of DexMa hydrogels are reported in Figure 3D–F. When prepared with ratios of 10:1 and 2:1, DexMa-TMPTA and DexMa-PETA hydrogels exhibit tan(delta) ≥ 0.1, comparable to the systems without crosslinkers. On the other hand, DexMa_10_-NPGDA_1_ and DexMa_2_-NPGDA_1_ showed a value of 0.065 after 1 or 5 min of crosslinking, presenting the characteristics of stronger hydrogels. Finally, adding the crosslinkers in a 4:1 ratio results in tan(delta) values lower than 0.1 for DexMa_4_-TMPTA_1_ and DexMa_4_-PETA_1_, for both irradiation times.

These interesting results make the platform extremely versatile because the resulting mechanical properties can be modulated by varying the type of crosslinker used and its concentration. Furthermore, compared to other systems described in the literature, the use of external methacrylate crosslinkers allows for the obtainment of systems with similar mechanical features but at lower Dex concentrations and/or, most importantly, lower crosslinkers concentrations, which might make the platform more biocompatible and safer to be used [60,61].

### 2.4. Semi-IPN Dynamic Mechanical Characterization

Due to these interesting results, the influence of the crosslinkers on DexMa network formation was also evaluated in a more complex system, in which Ge chains are interpenetrated into the crosslinked DexMa matrix. DexMa/Ge, DexMa-NPGDA/Ge, DexMa-TMPTA/Ge, and DexMa-PETA/Ge semi-IPNs were characterized via dynamic mechanical (penetration experiments) and rheological analyses (frequency sweep tests) to obtain information about stiffness, rigidity, and resistance of such matrices.

**Figure 4:**
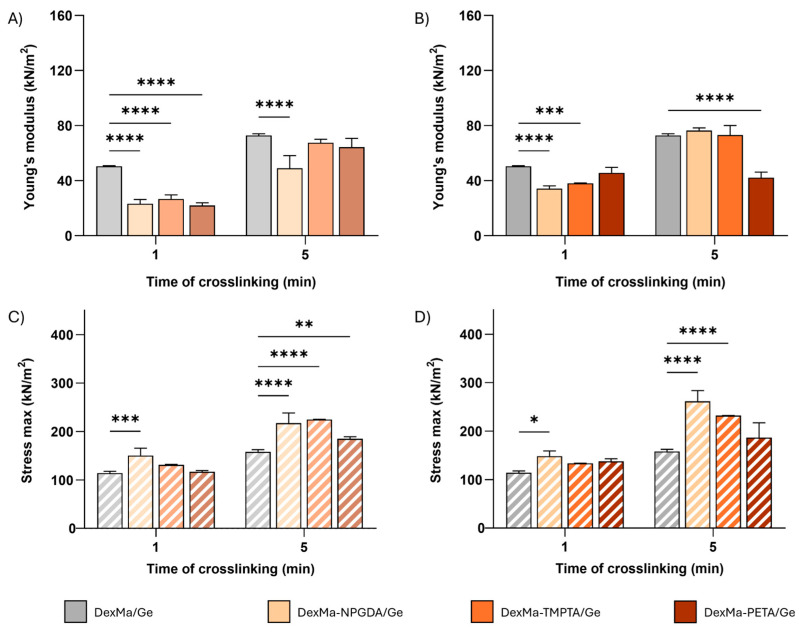
Dynamic mechanical analysis of DexMa/Ge semi-IPNs. Young’s modulus [E] of DexMa/Ge, DexMa-NPGDA/Ge, DexMa-TMPTA/Ge, and DexMa-PETA/Ge semi-IPNs obtained with a 4:1 (**A**) and 2:1 (**B**) ratio. Maximum stress registered at the breaking point of DexMa/Ge, DexMa-NPGDA/Ge, DexMa-TMPTA/Ge, and DexMa-PETA/Ge semi-IPNs in a ratio of 4:1 (**C**) and 2:1 (**D**). Data are expressed as the mean value ± standard deviation; experiments were performed in triplicate (*n* = 3). *p* values were obtained via two-way ANOVA and Dunnett’s multiple comparisons test (**** *p* < 0.0001; *** *p* < 0.001; ** *p* < 0.01; * *p* < 0.05).

In Figure 4, Young moduli (E) and the maximum stress sustained before breakage are reported for 4:1 and 2:1 crosslinker ratios. The lower ratio between the moles of methacrylic groups of dextran and the moles of crosslinkers was not investigated since no significative results were obtained on DexMa hydrogels. However, the E increase showed before was not confirmed in the case of semi-IPNs. In fact, by comparing the E values of DexMa_4_-NPGDA_1_/Ge, DexMa_4_-TMTPA_1_/Ge, and DexMa_4_-PETA_1_/Ge with the ones of DexMa/Ge (Figure 4A), it is clear that the added crosslinkers not only do not contribute to the rigidity of the system but result in a collapse of their mechanical features after one minute of crosslinking. Increasing the photocrosslinking time to 5 min, DexMa_4_-TMTPA_1_/Ge and DexMa_4_-PETA_1_/Ge performed better, displaying E values comparable to the control (∼70 kPa), while the introduction of a bivalent crosslinker seems to be detrimental. One possible explanation of this phenomenon could be that in the presence of Ge, which contributes to increase the overall viscosity of the system, crosslinkers might take longer to distribute homogeneously in the polymer mixture. A similar behavior was observed for the semi-IPNs with a ratio of 2:1 (Figure 4B): after one minute of crosslinking, DexMa_2_-NPGDA_1_/Ge, DexMa_2_-TMTPA_1_/Ge, and DexMa_2_-PETA_1_/Ge are less rigid than the control, confirming that such a small time of irradiation is not enough to facilitate the formation of intra- or inter-chain bonds, which is probably due to the increased viscosity and slowing of the radical species that are formed in the process. However, a longer exposure to the UV light allows the systems prepared with the bivalent and trivalent crosslinkers to act at least like the control, confirming that the presence of Ge exerts a levelling effect, strengthening the elastic component of the system.

Looking at the force registered right before the semi-IPN breaking point, the system’s maximum stress tolerance was obtained. In contrast to what was observed with the Young’s modulus, DexMa_4_-NPGDA_1_/Ge crosslinked for 1 and 5 min and DexMa_4_-TMPTA_1_/Ge crosslinked for 5 min are able to withstand higher stress than the respective controls (Figure 4C). On the other hand, both 1 and 5 min crosslinked DexMa_4_-PETA_1_/Ge do not show any significant difference compared to DexMa/Ge semi-IPN. The introduction of crosslinkers in a 2:1 ratio (Figure 4D) does not impact the resistance of the matrix after 1 min of irradiation, while, after 5 min of crosslinking, a decreasing trend can be observed as the functionality of the introduced crosslinkers increases. DexMa_2_-NPGDA_1_/Ge, DexMa_2_-TMPTA_1_/Ge, and DexMa_2_-PETA_1_/Ge are able to bear a stress of 260, 230, and 190 kN/m^2^, respectively, without mechanical degradation.

In Table 2, the percentage of deformation at which the gel rupture occurs is also reported. All semi-IPNs showed similar percentage values to those of the reference systems, except for DexMa_2_-PETA_1_/Ge, which can resist a deformation of more than 70%.

### 2.5. Semi-IPN Rheological Analysis

Then, DexMa/Ge semi-IPNs systems were also characterized via frequency sweep analysis, performed under stress-controlled conditions (Figure S3 in Supplementary Materials). As a result, all the systems showed a gel-like behavior (G′ > G″), and G′ values at 1 Hz were extrapolated. The analyses were performed in triplicate, after 1 or 5 min of UV irradiation, on DexMa-NPGDA/Ge, DexMa-TMPTA/Ge, and DexMa-PETA/Ge prepared with 4:1 and 2:1 ratios.

**Figure 5:**
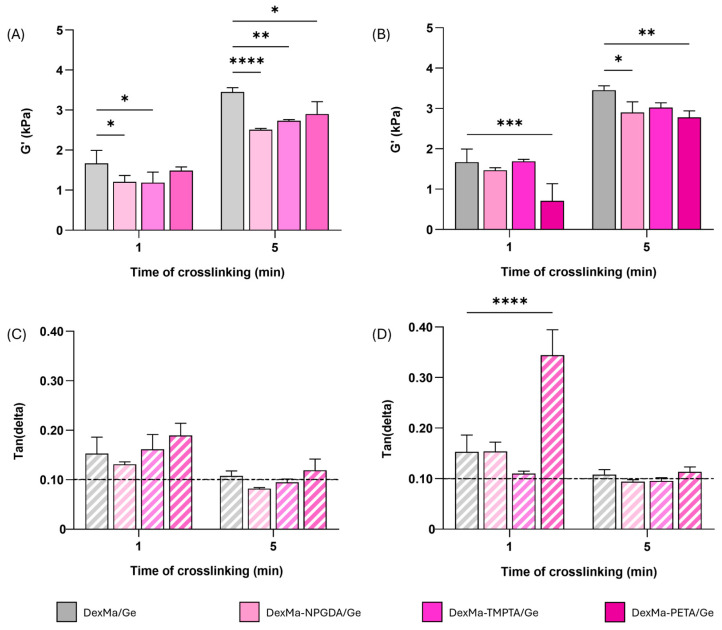
Semi-IPN rheological analysis, showing the shear storage modulus (G′) at a frequency of 1 Hz of DexMa/Ge, DexMa-NPGDA/Ge, DexMa-TMPTA/Ge, and DexMa-PETA/Ge obtained with a molar ratio of 4:1 (**A**) and 2:1 (**B**). Tan(delta) loss factor (G″/G′) at 1 Hz of semi-IPN obtained with a ratio of 4:1 (**C**) and 2:1 (**D**). Data are expressed as the mean value ± standard deviation; experiments were performed in triplicate (*n* = 3). *p* values were obtained via two-way ANOVA and Dunnett’s multiple comparisons test (**** *p* < 0.0001; *** *p* < 0.001; ** *p* < 0.01; * *p* < 0.05).

Independently from the time of exposure to UV light, adding crosslinkers in a 4:1 ratio resulted in a decrease in G′, leading to weaker systems compared to the control (Figure 5A). On the other hand, DexMa_2_-NPGDA_1_/Ge and DexMa_2_-TMPTA_1_/Ge semi-IPNs, after one minute of crosslinking, showed G′ values of 1.5 and 1.7 kPa, respectively, which is comparable to the reference. The use of the tetraacrylate crosslinkers, instead, does not bring any improvement in semi-IPNs’ mechanical properties (Figure 5B). Finally, five-minute crosslinking of DexMa_2_-NPGDA_1_/Ge, DexMa_2_-TMPTA_1_/Ge, and DexMa_2_-PETA_1_/Ge led to semi-IPN with a G′ value around 3 kPa, slightly lower than the DexMa/Ge sample. This result, in line with the dynamic mechanical characterization, further supports the hypothesis that the high viscosity of the polymer mixture may hinder both the crosslinker distribution and the movement of the radical species responsible for the crosslinking.

Furthermore, by looking at the G′ and G″ trend as a function of frequency, the moduli values at 1 Hz were extrapolated and the tan(delta) parameter was calculated as the G″/G′ ratio. After 1 min of crosslinking, all the systems prepared with a 4:1 ratio showed a tan(delta) > 0.1, as well as the one used as reference (Figure 5C). The increase in the crosslinking time led to a decrease in the tan(delta), with DexMa_4_-NPGDA_1_/Ge and DexMa_4_-TMPTA_1_/Ge values equal to 0.08 and 0.09, respectively, meaning that a higher exposition to UV light is needed to obtain stronger semi-IPNs. The same scenario was observed in the matrices fabricated with a 2:1 ratio, where 1 min of crosslinking seems to be insufficient for the stiffening of the material (Figure 5D). On the other hand, a longer irradiation results in stronger DexMa_2_-NPGDA_1_/Ge and DexMa_2_-TMPTA_1_/Ge semi-IPNs, with respect to the system without a crosslinker.

Overall, the diacrylate and triacrylate crosslinkers seems to perform better than PETA at higher irradiation times, leading to matrices that at least have similar or better mechanical properties than the one without crosslinkers. For this reason, DexMa_2_-NPGDA_1_/Ge and DexMa_2_-TMPTA_1_/Ge semi-IPNs, crosslinked for 5 min, were further investigated after physical crosslinking of Ge chains.

### 2.6. IPN Formation: Ge Physical Crosslinking

Ge is an anionic polysaccharide that can undergo a sol–gel transition (a so called ionotropic gelation), in the presence of cations, which results in the formation of physically crosslinked matrices [62]. Complexation of divalent cations leads to particularly strong hydrogels through the aggregation of helixes, while the addition of monovalent cations produces weaker hydrogels due to electrostatic interactions with the polysaccharide’s carboxylate groups [63]. In this light, such an interesting property has been exploited to increase the mechanical resistance and performances of DexMa/Ge semi-IPNs, by exchanging glycerol with HEPES buffer solution, rich in Ca^2+^ ions, after crosslinking of the systems. In fact, the choice of glycerol as a solvent remains extremely convenient to promote the rapid crosslinking of DexMa chains [32], but the subsequent inclusion of Ca^2+^ ions in the matrix can lead to the physical crosslinking of the Ge chains, which are interspersed into the chemically crosslinked DexMa matrix. In this way, the system is converted from a semi-IPN into an IPN, in which the two polysaccharides are crosslinked independently of each other. Moreover, glycerol itself is not biologically inert. In fact, if it is present at a concentration higher than 10–15%, it causes cell cycle arrest, probably because of irreversible disruption of membrane permeability and alteration of intracellular ions [64]. Therefore, the solvent exchange was necessary to make the material biocompatible and suitable to be used as a scaffold for different biomedical applications.

The replacement of glycerol was carried out following the procedure reported in Section 4.2.4, in such a way that Ca^2+^ is introduced progressively and interacts gradually with the carboxyl groups of Ge. A final washing step in HEPES buffer was needed to remove the unbound excess of calcium ions. To confirm that most of the glycerol was removed from the systems, it was derivatized into a molecule visible in the UV-vis range and then quantified via HPLC. In detail, via the addition of sodium periodate, glycerol is oxidized to formaldehyde, which in the presence of ammonia can react with two molecules of acetylacetone to yield 3,5-diacetyl-1,4-dihydrolutidine [56,65].

The residual glycerol within each IPN was calculated by subtracting the amounts of glycerol removed in each step from the initial weight. The amounts of DexMa, Ge, NPGDA, TMPTA, or PETA were subtracted from the obtained value. The percentage of residual glycerol was less than 5%, which is compatible with its use in biomedical applications [66,67].

#### Calcium Quantification

To be sure that the amount of Ca^2+^ ions is enough to ensure a homogeneous gelation of the system, a quantification of the cation was needed. The maximum strength of Ge hydrogel with divalent cations comes at about stoichiometric equivalence to the Ge carboxylate groups [68]. Therefore, a direct quantification was performed following the procedure reported in Section 4.2.6. First, samples containing organic material required a preliminary treatment before metal analysis. A mixture of concentrated acids was used to digest the polymeric matrix, dissolving metals in soluble salt forms. The metals were then quantified measuring the optical emission produced by the liquid sample when introduced into an argon gas plasma, inductively coupled. This technique allows the determination of the metals in the matrix, in our case calcium, in a wide range of concentrations.

Before experimentally determining the amount of calcium within the system, a theoretical calculation was made considering that calcium binds the carboxyl groups of Ge, present in the IPN at a concentration of 1% *w*/*w*. Since Ca^2+^ is a bivalent cation, we can assume that each ion is complexed by two carboxyl groups, corresponding to 31 g of Ca^2+^/gram of Ge. As reported in Table 3, an amount of Ca^2+^ ions about 4.5-times higher than expected was found in samples A and B, meaning that an extra purification step might be required. Thus, a fifth washing step with HEPES buffer solution was carried out, and the matrix was treated as described before (sample C). The analysis showed a calcium amount comparable to the theoretical value, meaning that the excess of Ca^2+^ has been successfully removed.

### 2.7. IPN Characterization

**Figure 6:**
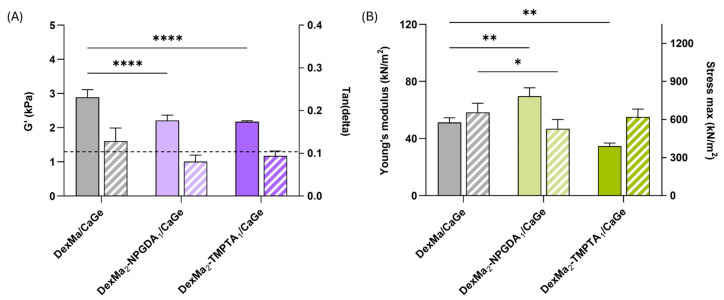
Rheological analysis of DexMa/CaGe, DexMa-NPGDA/CaGe, and DexMa-TMPTA/CaGe IPNs obtained with a 2:1 ratio (**A**), showing the shear storage modulus (G′) at a frequency of 1 Hz (full bars) and tan(delta) loss factor (G″/G′) at 1 Hz (striped bars). Dynamic mechanical analysis (**B**): Young’s modulus [E] of DexMa/CaGe, DexMa-NPGDA/CaGe, and DexMa-TMPTA/CaGe IPNs obtained with a 2:1 ratio (full bars) and maximum stress registered at the breaking point (striped bars). Data are expressed as the mean value ± standard deviation; experiments were performed in triplicate (*n* = 3). *p* values were obtained via two-way ANOVA and Dunnett’s multiple comparisons test (**** *p* < 0.0001; ** *p* < 0.01; * *p* < 0.05).

The stiffness of DexMa/CaGe, DexMa_2_-NPGDA_1_/CaGe, and DexMa_2_-TMPTA_1_/CaGe IPNs was evaluated after 5 min of crosslinking, using the same parameters and techniques reported for hydrogels and semi-IPNs analyses, to assess if the presence of the diacrylate and triacrylate crosslinkers could affect the properties of the final IPN system. As reported in Figure 6A (full bars), rheological experiments showed that the G′ values recorded at 1 Hz of IPNs with crosslinkers are lower than the reference, confirming the idea that both the distribution of crosslinkers and the movement of the radical species during the crosslinking may be hindered by the high viscosity of the polymer mixture. However, looking at the tan(delta) parameter (Figure 6A—striped bars), both DexMa_2_-NPGDA_1_/CaGe and DexMa_2_-TMPTA_1_/CaGe IPNs had a tan(delta) < 0.1, lower than the control. Therefore, the addition of NPGDA and TMPTA contribute to the formation of stronger gels (Full G′/G″ curves are reported in Figure S4 in Supplementary Materials).

Moreover, even though there is no difference in terms of stress tolerance (Figure 6B—stiped bars), a significant advantage in the use of the diacrylate crosslinker has been highlighted following compression analysis: DexMa_2_NPGDA_1_/CaGe IPN exhibit an E value of 70 kN/m^2^, respectively, 1.4- and 2-fold higher than the ones of DexMa/CaGe and DexMa_2_-TMPTA_1_/CaGe IPNs (Figure 6B—full bars). This increase has not been observed in the semi-IPNs, where DexMa_2_-NPGDA_1_/Ge and DexMa_2_-TMPTA_1_/Ge acted like the control (Figure 4B). In this case, we can suppose that a crosslinker with higher functionality (i.e., TMPTA) leads to a higher crosslinking density that might hinder the physical crosslinking of Ge chains with Ca^2+^ ions. On the other hand, using NPGDA might be a compromise that results in a stable and strong gel, both chemically and physically crosslinked.

It should also be pointed out that, compared to other dextran-based IPN systems already described [69,70], the introduction of multivalent acrylate crosslinkers result in platforms with increased stiffness and resistance to penetration, characteristics that make these matrices suitable for applications where scaffolds need to withstand greater stress. This helps broaden the range of applicability of these materials in the current biomedical landscape.

## 3. Conclusions

In recent years, biomaterials have significantly enhanced quality of life, paving the way for advanced medical solutions. Due to their biocompatibility and soft structure resembling living tissues, polysaccharide-based hydrogels are generating increasing interest in the drug delivery and tissue engineering fields. However, they often lack suitable mechanical properties which restricts their use. Several strategies have been exploited to improve the stiffness of polysaccharide-based scaffolds, including the design of multicomponent networks (semi-IPN and IPN) and the introduction of external crosslinkers. In this context, our study investigated the introduction of methacrylate crosslinkers with different functionalities in dextran-based, single-network hydrogel, and double-network IPN systems. Dex was modified via the introduction of methacrylic groups (DexMa) and crosslinked with UV light in the presence of different concentrations of crosslinkers: neopentyl glycol diacrylate (NPGDA), trimethylolpropane triacrylate (TMPTA), and pentaerythritol tetraacrylate (PETA). As a result, the addition of crosslinkers led to stronger hydrogels, with a better resistance to penetration and breaking than the control. Based on these findings, these molecules were also evaluated on the Dex/gellan semi-IPN (DexMa/Ge). However, the systems showed similar mechanical properties as the one without crosslinkers. Finally, after a solvent exchange and the introduction of Ca^2+^ ions, semi-IPNs were turned into IPN systems (DexMa/CaGe) via Ge ionotropic gelation. In this case, DexMa-NPGDA/CaGe IPN performed better than the others, suggesting that a different crosslinking density, related to different crosslinking functionality, might influence Ge gelation. These findings suggest that by varying the crosslinker, the concentration, and the time of irradiation, it is possible to modulate the mechanical properties of polysaccharide-based hydrogels, semi-IPN, and IPN, expanding their potential and future applications.

## 4. Materials and Methods

### 4.1. Materials

Dextran from Leuconostoc sep. (Dex; Mw = 40 × 10^3^), 4-N,N-dimethylaminopyridine (DMAP), glycidyl methacrylate (GMA), 2-hydroxy-4′-(2-hydroxy-etoxy)-2-methyl-propriophenone (Irgacure 2959), glycerol, neopentyl glycol diacrylate (NPGDA), trimethylolpropane triacrylate (TMPTA), pentaerythritol tetraacrylate (PETA), N-(2-hydroxyethyl)-piperazine-N’-(2-ethanesulfonic acid) (HEPES), ethanol (EtOH), calcium chloride, sodium metaperiodate, acetylacetone, ammonium acetate, acetic acid, acetonitrile (CH_3_CN), and dimethyl sulfoxide (DMSO) were provided by Sigma-Aldrich (Milano, Italy). Low acyl gellan gum sodium salt (Ge; Mw = 2.5 × 10^6^) was purchased from CP Kelco (San-Diego, CA, USA). All chemicals were received and used without further purification.

### 4.2. Methods

#### 4.2.1. Synthesis of Dextran Methacrylate (DexMa)

DexMa synthesis was carried out as previously described [71,72]. Briefly, a solution of Dex in anhydrous DMSO 100 mg/mL was prepared at room temperature, and then, 200 mg DMAP and 174 μL of GMA were added per gram of polysaccharide, to obtain a degree of substitution (DS) of 20% mol/mol (mol of methacrylate groups per mol of Dex repeating units). After 48 h of magnetic stirring at room temperature, the pH was neutralized with HCl 1 M, and the product was dialyzed against double-distilled water (cut off 12,000–14,000) until a conductivity < 2 mS was reached. The final derivative was recovered via freeze-drying and stored in a desiccator protected from light (yield 90–95%). ^1^H-NMR analysis in D_2_O were recorded with a Bruker (Billerica, MA, USA) Avance 400 (Milano, Italy) spectrometer to assess the real degree of methacrylation.

#### 4.2.2. Preparation of DexMa-NPGDA, DexMa-TMPTA, and DexMa-PETA Hydrogels

First, neopentyl glycol diacrylate (NPGDA), trimethylolpropane triacrylate (TMPTA), and pentaerythritol tetraacrylate (PETA) were solubilized in DMSO at different concentrations to test different molar ratio between the moles of methacrylic group on the dextran and the moles of crosslinkers, as reported in Table 4. Then, 342 mg of DexMa were solubilized in 5.7 g of glycerol at 80 °C for 15 min (concentration 5% *w*/*w*) and 777 μL of each solution of crosslinkers were added dropwise under stirring. After 3 min, 83 μL of an 8 w/V Irgacure 2959 solution was added, and the mixture was gently stirred for another 2 min at 80 °C. The polymeric solution was transferred still hot into cylindric plastic molds of 20 mm in diameter (1.5 g per mold), and they were left to cool for 2 h at RT. To protect the samples from the UV light action, all the samples were stored in the dark. Finally, DexMa-NPGDA, DexMa-TMPTA, and DexMa-PETA hydrogels were obtained via photocrosslinking, using a mercury UV lamp (λ = 356 nm; I = 20 mW/cm^2^), for 1 and 5 min at a distance of 4 cm. As a control, DexMa hydrogels were prepared following the same procedure, but by adding 777 μL of pure DMSO without crosslinkers.

#### 4.2.3. Preparation of DexMa-NPGDA/Ge, DexMa-TMPTA/Ge, and DexMa-PETA/Ge Semi-IPNs

A 10% *w*/*w* DexMa solution was prepared by solving 485 mg of functionalized polymer in 3.8 g of glycerol at 80 °C for 20 min. Similarly, 100 mg of Ge were solubilized in 4.9 g of glycerol at 100 °C for 2 h to obtain a 2% *w*/*w* solution. After complete dissolution, 518 μL of NPGDA, TMPTA, or PETA in DMSO (see Table 1) were added dropwise to DexMa solution, under stirring. Then, the two polymer solutions were merged in equal parts and stirred at 80 °C until complete homogeneity. Finally, 12 μL of an 8% w/V Irgacure 2959 solution was added for each gram of the polymer mixture, and the solution was gently stirred for 1 min at 80 °C. Aliquots of 1.5 g were transferred in cylindric plastic molds (diameter × height = 20 mm × 10 mm) and stored at RT for 2 h sheltered from light. DexMa-NPGDA/Ge, DexMa-TMPTA/Ge, and DexMa-PETA/Ge semi-IPNs were obtained by irradiating the samples, using a mercury UV lamp (λ = 356 nm; I = 20 mW/cm^2^), for 1 and 5 min at a distance of 4 cm. As a control, DexMa/Ge samples were prepared following the same procedure, but by adding 518 μL of pure DMSO, without crosslinkers.

#### 4.2.4. IPN Formation and Quantification of Removed Glycerol

The glycerol present in DexMa/Ge, DexMa-NPGDA/Ge, DexMa-TMPTA/Ge, and DexMa-PETA/Ge semi-IPNs was replaced with an aqueous medium containing Ca^2+^ ions [56]. After the polymeric mixture was prepared (Section 4.2.3), aliquots of 0.75 g were transferred in cylindric plastic molds and crosslinked to obtain the semi-IPNs. Then, four extractions were performed under gentle magnetic stirring for 30 min with 220 mL of (I) EtOH 96% *V*/*V*; (II) a CaCl_2_ solution 1 M in EtOH; (III) a mixture of CaCl_2_ in EtOH 1 M: HEPES 0.05 M (pH 7.4) 50:50; and (IV) a mixture of EtOH 96% *V*/*V*: HEPES 0.05 M (pH 7.4) 50:50. The purified DexMa/CaGe IPNs were transferred into Petri dishes for further analysis, while the extraction liquids were collected to quantify the removed glycerol. An indirect quantification was performed by using a Knauer Azura HPLC system (Knauer, Berlin, Germany) equipped with a binary pump (Azura P6.1L) and a UV-Vis detector (190–750 nm, Azura UDV 2.1L), controlled with Clarity software (v. 6.2.0.208). Samples were injected into a Knauer Eurospher II C18 (Knauer, Berlin, Germany) column (5 μm, 4.6 x 250 mm) with CH_3_CN (+0.1% *V*/*V* of TFA): H_2_O (+0.1% *V*/*V* of TFA) at a ratio of 30:70 to 100:0 as eluent, at a flow of 1 mL/min. However, since glycerol does not absorb in the UV range, the molecule was converted before each analysis into 3,5-diacetyl-1,4-dihydrolutidine, a chromophore with an absorption peak at 410 nm. After preparing an acetate buffer at pH 5.5 by mixing an equal volume of acetic acid 1.6 M and ammonium acetate 4.0 M, a solution of sodium periodate 10 mM (reagent A) and acetylacetone 0.2 M (reagent B) were prepared daily before the HPLC analysis. The first two extraction liquids (I–II) were merged, and the reaction was carried out by diluting 1 mL of them with 1 mL of HEPES 0.05 M, followed by the addition of 1.2 mL of reagent A. After 30 sec of gentle shaking, 1.2 mL of reagent B was added, and the mixture was heated at 70 °C for 1 min under magnetic stirring. At this point, the mixture was immediately cooled at 20 °C to stop the reaction and analyzed via HPLC after 3 min. The same workflow was applied to 2 mL of the last two extraction liquids (III–IV), without any starting dilution. Indirect quantification was performed using a calibration curve previously determined with glycerol standard solutions in the range of 6.25–400 μg/mL (R^2^ = 0.999, *n* = 7).

#### 4.2.5. Rheological and Dynamic Mechanical Characterization of Hydrogels, Semi-IPNs, and IPNs

Rheological measurements were performed using a stress-controlled Discovery Hybrid Reometer-1 (TA Instruments—New Castle, DE, USA) equipped with a Peltier temperature controlling unit. Amplitude sweep experiments were performed by applying a fixed frequency of 1 Hz in the range of 0.01–100 Pa, to determine the linear viscoelastic (LVE) region. Frequency sweep analyses were run from 50 to 0.01 Hz, by applying a constant strain (γ) of 1%. All the measurements were conducted in triplicate at 25 °C, using a grained parallel plate geometry (diameter = 20 mm). Mechanical properties of the systems were also investigated using a TA-XT2i Texture Analyser (Stable Micro Systems, Surrey, UK), controlled with Texture Expert Exceed software (v. 2.03), and equipped with a 5 kg load cell. Samples were penetrated with an ebonite cylindric probe (P10; diameter = 10 mm) by setting pretest, test, and post-test speeds of 2.0 mm/s, 1.0 mm/s and 2.0 mm/s, respectively. After setting a trigger force of 0.01 N and a maximum distance of 4 mm (80% of the sample’s height), the force needed for the penetration (stress) was recorded as a function of the distance covered by the probe (strain); Young’s modulus (E) was calculated as the slope of the curve in the strain range of 0–10%. The experiments were carried out in triplicate at 25 °C.

#### 4.2.6. Quantification of Ca^2+^ Ions

For the quantification of Ca^2+^ ions, DexMa/CaGe IPNs were dried, weighed, and then transferred to a suitable vessel. After adding 10 mL of reverse aqua regia (HNO_3_:HCl 1:3), the vessel was hermetically sealed, and the matrix was digested in a microwave for 85 min. In particular, a first temperature ramp from 20 °C to 200 °C was set for 25 min, followed by a second temperature ramp up to 240 °C for 30 min, and, finally, a final cooling time of 30 min ended the process. The solution was then transferred to a 50 mL tube and brought to the mark with water. The solution was then sonicated for 15 min and centrifuged for 5 min at 7500 rpm. Finally, an aliquot of this solution was analyzed via ICP OES (Inductively Coupled Plasma-Optical Emission Spectroscopy, Thermo Fisher Scientific, Waltham, MA, USA) to determine the amount of calcium present within the polymeric matrix.

### 4.3. Statistical Analyses

Statistical analyses were performed using the software GraphPad Prism V8.0 (La Jolla, CA, USA). The fold change in mechanical strength was assessed via two-way ANOVA, followed by Dunnett’s multiple comparison test. *p* values are reported on the graphs.

## Figures and Tables

**Table 1:** Percentage of deformation of DexMa, DexMa-NPGDA, DexMa-TMPTA, and DexMa-PETA hydrogels before breaking.

Ratio	Hydrogel	Percentage of Deformation After 1′ of Crosslinking (%)	Percentage of Deformation After 5′ of Crosslinking (%)
	DexMa	44.1 ± 0.2	47.7 ± 0.8
10:1	DexMa_10_-NPGDA_1_	56.4 ± 0.1	57.7 ± 0.4
	DexMa_10_-TMPTA_1_	43.0 ± 2.3	47.0 ± 0.5
	DexMa_10_-PETA_1_	44.2 ± 0.3	46.8 ± 1.5
4:1	DexMa_4_-NPGDA_1_	59.2 ± 1.7	56.4 ± 1.3
	DexMa_4_-TMPTA_1_	46.4 ± 1.4	45.0 ± 1.0
	DexMa_4_-PETA_1_	44.8 ± 0.8	45.5 ± 1.8
2:1	DexMa_2_-NPGDA_1_	56.1 ± 0.7	55.1 ± 1.4
	DexMa_2_-TMPTA_1_	42.9 ± 0.9	43.9 ± 0.7
	DexM_2_a-PETA_1_	42.6 ± 0.5	42.0 ± 1.0

**Table 2:** Percentage of deformation of DexMa/Ge, DexMa-NPGDA/Ge, DexMa-TMPTA/Ge, and DexMa-PETA/Ge semi-IPNs at breaking point.

Ratio	Hydrogel	Percentage of Deformation After 1′ of Crosslinking (%)	Percentage of Deformation After 5′ of Crosslinking (%)
	DexMa/Ge	63.4 ± 4.8	63.6 ± 3.2
4:1	DexMa_4_-NPGDA_1_/Ge	68.3 ± 2.8	62.9 ± 2.1
	DexMa_4_-TMPTA_1_/Ge	66.3 ± 0.8	59.7 ± 1.0
	DexMa_4_-PETA_1_/Ge	68.8 ± 0.5	68.1 ± 6.9
2:1	DexMa_2_-NPGDA_1_/Ge	65.6 ± 1.5	61.5 ± 1.8
	DexMa_2_TMPTA_1_/Ge	63.3 ± 2.0	61.3 ± 1.8
	DexMa_2_PETA_1_/Ge	72.4 ± 8.0	70.4 ± 2.9

**Table 3:** Ca^2+^ quantification via ICP OES within DexMa/CaGe systems after 4 (A1–2 and B1–2) and 5 washing steps (C1–2). Results are expressed as the mean value ± standard deviation; experiments were performed in duplicate (*n* = 2).

Sample	Ca (ppm) 393.366 Radial	Ca (mg)	Ca (mg/g of Ge-Na^+^)
A1	19.88 ± 0.25	0.99 ± 0.01	147.29 ± 1.82
A2	21.40 ± 0.28	1.07 ± 0.01	158.50 ± 2.11
B1	14.58 ± 0.08	0.73 ± 0.01	124.63 ± 0.67
B2	14.51 ± 0.15	0.73 ± 0.01	123.97 ± 1.24
C1	2.63 ± 0.01	0.13 ± 0.01	25.87 ± 0.01
C2	2.79 ± 0.02	0.14 ± 0.01	27.43 ± 0.22

**Table 4:** Concentration of NPGDA, TMPTA, and PETA in DMSO to test 10:1, 4:1, and 2:1 molar ratios.

Molar Ratio	NPGDA (mg/mL)	TMPTA (mg/mL)	PETA (mg/mL)
10:1	42	59	70
4:1	104	145	173
2:1	208	290	345

## Data Availability

The original contributions presented in this study are included in the article/Supplementary Materials. Further inquiries can be directed to the corresponding author.

## Supplementary Materials

The following supporting information can be downloaded at https://www.mdpi.com/article/10.3390/gels10120773/s1: Figure S1: ^1^H-NMR spectra of Dex and DexMa in which the most relevant peaks and integrations are highlighted; Figure S2: Rheological analysis of DexMa, DexMa-NPGDA, DexMa-TMPTA, and DexMa-PETA hydrogels, showing the shear storage and loss moduli trends as a function of frequency; Figure S3: Rheological analysis of DexMa/Ge, DexMa-NPGDA/Ge, DexMa-TMPTA/Ge, and DexMa-PETA/Ge semi-IPN, showing the shear storage and loss moduli trends as a function of frequency; Figure S4: Rheological analysis of DexMa/CaGe, DexMa_2_-NPGDA_1_/CaGe, and DexMa_2_-TMPTA_1_/CaGe IPN, showing the shear storage and loss moduli trends as a function of frequency.
