# Supplementary material for: Reinforcement of Dextran Methacrylate-Based Hydrogel, Semi-IPN, and IPN with Multivalent Crosslinkers
Source: Gels. 2024 Nov 27;10(12):773. doi: 10.3390/gels10120773 (PMC11675718; doi:10.3390/gels10120773)
Supplement: Supplementary file 1 [file gels-10-00773-s001.zip › gels-3315540-supplementary.pdf]

Supporting Materials

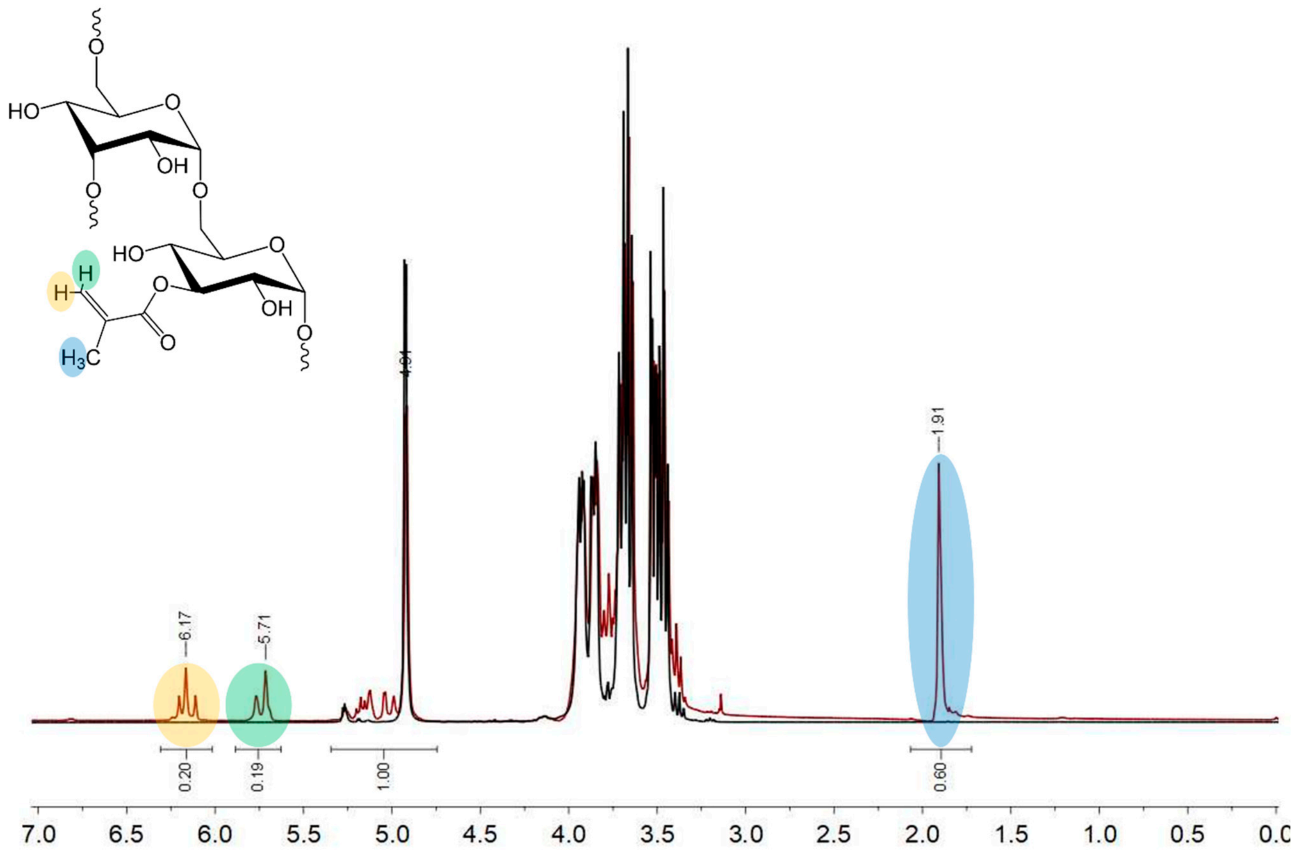

Figure S1. ^{1}H-NMR spectra of Dex (black) and DexMa (red) in which the most relevant peaks and integrations are highlighted.

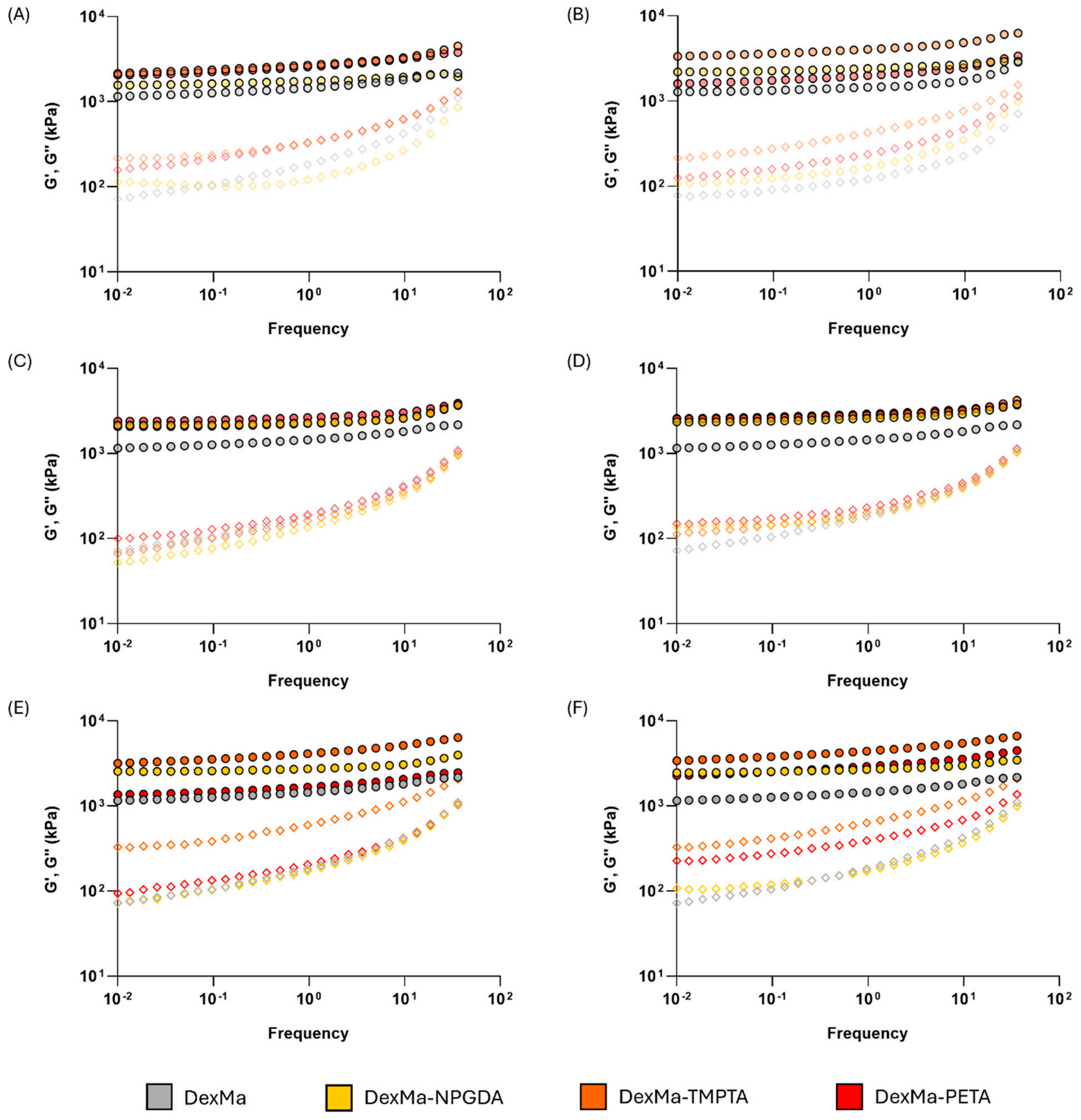

Figure S2. Rheological analysis of DexMa, DexMa-NPGDA, DexMa-TMPTA and DexMa-PETA hydrogels, showing the shear storage modulus (G′ – full circles) and loss modulus (G′′ – empty squares) trends as a function of frequency. Hydrogels were obtained using ratio 10:1, crosslinked for 1 (A) and 5 minutes (B), ratio 4:1, irradiated for 1 (C) and 5 minutes (D), and ratio 2:1, crosslinked for 1 (E) and 5 minutes (F).

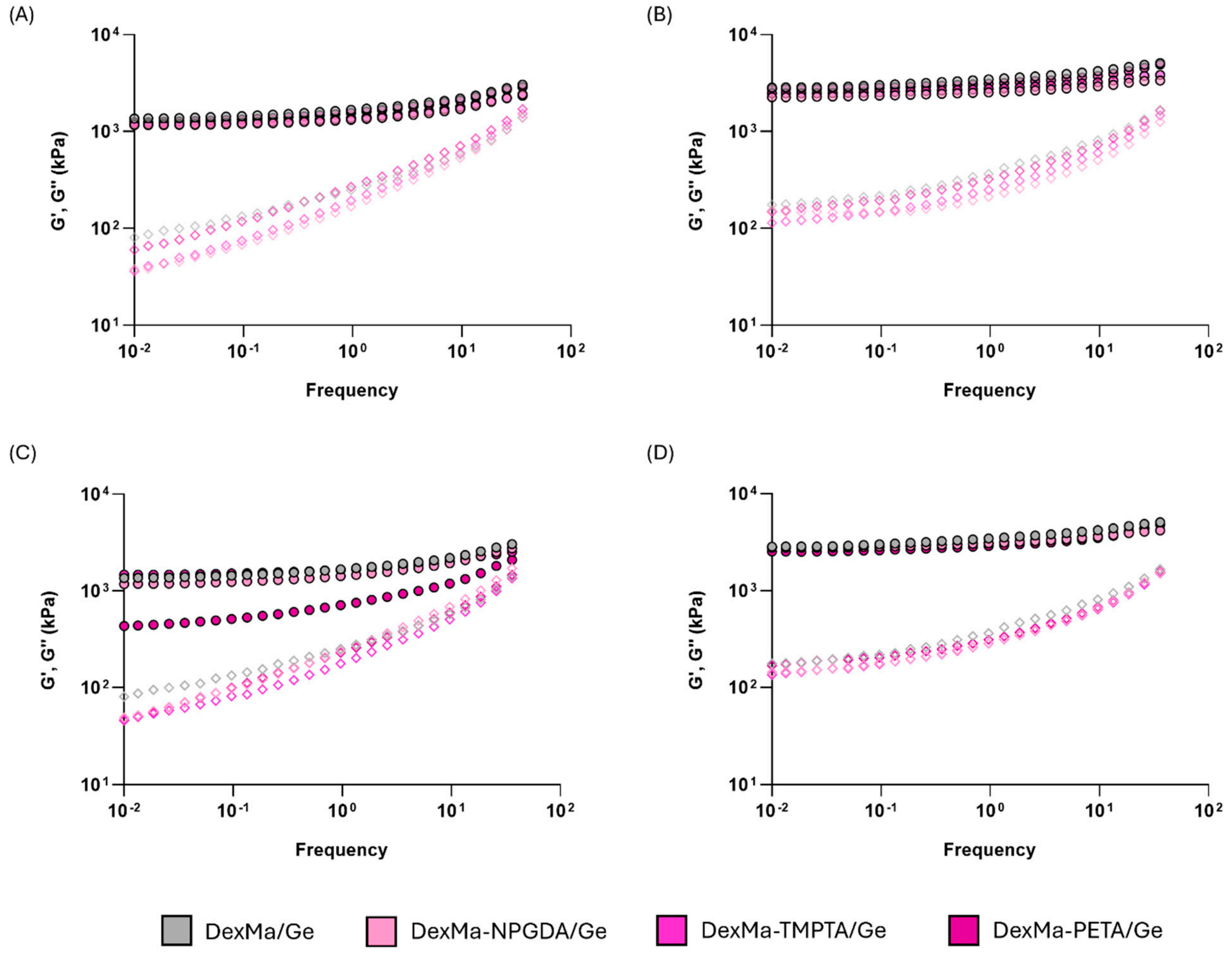

Figure S3. Rheological analysis of DexMa/Ge, DexMa-NPGDA/Ge, DexMa-TMPTA/Ge and DexMa-PETA/Ge semi-IPN, showing the shear storage modulus (G′ – full circles) and loss modulus (G′′ – empty squares) trends as a function of frequency. Semi-IPN were obtained using ratio 4:1, crosslinked for 1 (A) and 5 minutes (B), and ratio 2:1, crosslinked for 1 (C) and 5 minutes (D).

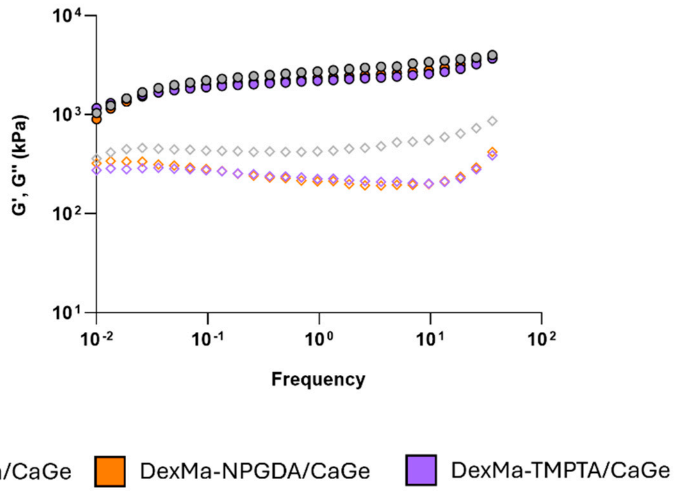

Figure S4. Rheological analysis of DexMa/CaGe, DexMa$_2$-NPGDA$_1$/CaGe and DexMa$_2$-TMPTA$_1$/CaGe IPN, showing the shear storage modulus (G′ – full circles) and loss modulus (G′′ – empty squares) trends as a function of frequency. IPN were obtained using ratio 2:1 and crosslinked for 5 minutes.
